# Infectious endarteritis in aortic coarctation: two spectra of an infrequent disease

**DOI:** 10.1590/1984-0462/2024/42/2023084

**Published:** 2023-12-22

**Authors:** Justo Santiago, Gabriela Karl, Claudia Florez, Yudisay Molina, Javier Castro, Alexandra Hurtado, Valeria García

**Affiliations:** a Cardiovascular Foundation of Colombia, Bucaramanga, Santander, Colombia.; b Santander University Hospital, Bucaramanga, Santander, Colombia

**Keywords:** Endarteritis, Aortic coarctation, Streptococcus mitis, Endarterite, Coartação aórtica, Streptococcus mitis

## Abstract

**Objective:**

To describe two different degrees of clinical commitment and results in the evolution of infectious endarteritis in patients without a previous diagnosis of aortic coarctation.

**Case description:**

Two male patients aged 13 and 9 years old were admitted. The first due to a fever for 2 months, which started after dental cleaning, and the second due to high blood pressure, both patients with asthenia and weight loss. In the first case, the transthoracic echocardiogram showed aortic coarctation, and the transesophageal echocardiogram showed the presence of vegetations in the post-coarctation area, without pseudoaneurysms, with blood culture positive for *Streptococcus mitis*. This patient was treated for six weeks with crystalline penicillin, resolving the infection without complications. The second case was assessed for high blood pressure with a history of fever, and was treated with antibiotics. When performing a transthoracic echocardiogram, aortic coarctation was observed with a saccular image classified as a pseudoaneurysm by angiography and tomography. Blood culture was negative, and the patient developed an episode of hematemesis whose initial etiology could not be determined. Before surgical repair, he had a second episode of copious hematemesis with hypovolemic shock and death.

**Comments:**

We need to have a high index of clinical suspicion to establish the diagnosis of aortic coarctation complicated by endarteritis and start the appropriate antibiotic treatment, always maintaining surveillance for the early detection of pseudoaneurysms.

## RESUMO

**Objetivo:**

Descrever dois diferentes graus de comprometimento clínico e resultados na evolução de endarterite infecciosa em pacientes sem diagnóstico prévio de coarctação da aorta.

**Descrição do caso:**

Dois pacientes do sexo masculino com idades entre 13 e nove anos foram internados. O primeiro por febre durante dois meses, iniciada após limpeza dentária. O segundo por hipertensão arterial. Ambos com astenia e perda de peso. No primeiro caso, o ecocardiograma transtorácico mostrou coarctação da aorta e o ecocardiograma transesofágico revelou vegetações na área pós-coarctação, sem pseudoaneurismas. A hemocultura foi positiva para de *Streptococcus mitis*. Este paciente foi tratado por seis semanas com penicilina cristalina, resolvendo a infecção sem complicações. O segundo caso foi avaliado pela presença de hipertensão arterial, com história de febre tratada com antibióticos. Ao realizar o ecocardiograma transtorácico, observou-se coarctação da aorta com imagem sacular classificada como pseudoaneurisma pela angiografia e tomografia. A hemocultura foi negativa. O paciente desenvolveu um episódio de hematêmese, cuja etiologia inicial não pôde ser determinada. Antes da correção cirúrgica, apresentou um segundo episódio de hematêmese profusa, com choque hipovolêmico e óbito.

**Comentários:**

Devemos ter um alto índice de suspeição clínica para poder estabelecer o diagnóstico de coarctação da aorta complicada com endarterite e iniciar o tratamento antibiótico adequado. É preciso manter a vigilância para a detecção precoce de pseudoaneurismas.

## INTRODUCTION

Infectious endarteritis (IE) is a serious and rare presentation of aortic coarctation, since most patients are diagnosed and treated early. Before developing antibiotics and surgical repair techniques, IE was responsible for 20% of deaths in patients with this congenital malformation.^1^ In cases of aortic infection, the most feared complication is the development of mycotic aneurysms, given the high morbidity and mortality they present.^2^

Two cases of patients without a previous diagnosis of aortic coarctation were presented here, with different degrees of involvement of the aorta, and, therefore, with different results.

## CASE REPORTS

### Case 1

**Figure 1:**
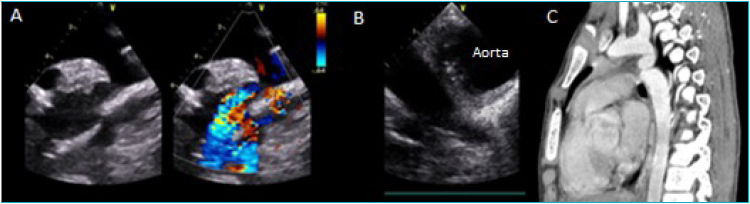
**A.** Transthoracic echocardiogram (TTE) Suprasternal view: aortic arch with an area of coarctation and post-stenotic dilation.**B.** Transesophageal echocardiogram (TEE) short-axis view (0°) of the descending aorta at the post-coarctation mid-esophageal level, where an image of vegetation can be seen. **C.** Angiotomography showing aortic coarctation.

A 13-year-old male from a rural area consulted an outpatient center with a 1-month history of fever accompanied by asthenia and weight loss. As an antecedent, dental cleaning was done seven days before the onset of the fever. Cytomegalovirus, Ebstein Barr, Herpesvirus, toxoplasmosis, HIV, dengue, and Chagas infections were ruled out. Blood cultures were negative, and the patient was referred to the oncology service for evaluation, A bone marrow aspirate ruled out lymphoproliferative syndrome. An echocardiogram was requested to rule out endocarditis. The echocardiogram found a coarctation of the aorta, without valvular or mural vegetation (Figure 1A). The angior sonance showed alteration in the thickness of the aortic walls at the level of the aortic arch with an area of coarctation, suggesting an inflammatory type of alteration. Takayasu disease was excluded. The angiotomography confirmed aortic coarctation without aneurysms (Figure 1C). Additionally, an area of splenic infarction was evidenced. The patient remained febrile and presented erythematous lesions in the nail beds of the lower limbs during fever peaks. He was referred to our institution after two months of febrile symptoms and was admitted with a weight of 39 kg, blood pressure in the right upper limb of 139/92 mm Hg, and 109/78 in the ipsilateral lower limb. He did not present murmurs on auscultation. Laboratory tests showed 12,000 leukocytes with 80% segmentation, C-reactive protein of 46.50 mg/L, and erythrocyte segmentation rate of 38 mm/second. The transesophageal echocardiogram showed a functional bicuspid aortic valve with mild aortic regurgitation, without any image suggestive of vegetation (Figure 1B). At the level of the descending aorta, the area of coarctation showed wall thickening after the obstruction with hyper-refringent images suggestive of 1-mm vegetations and without images of aneurysms. Two additional blood cultures showed growth of penicillin-sensitive Streptococcus mitis. A penicillin G scheme was administered for six weeks. The evolution was favorable with remission of fever, negative blood cultures, normalization of acute phase reactants, and weight recovery. Subsequently, the patient was taken for endovascular repair of the aortic coarctation.

### Case 2

**Figure 2:**
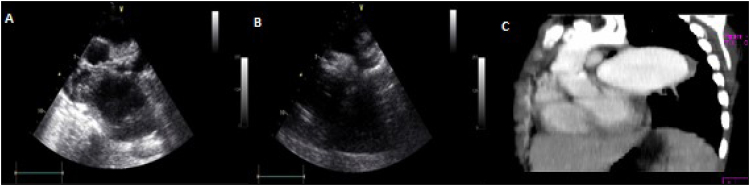
**A and B.** Echocardiogram in suprasternal view appreciating aortic arch with coarctation and saccular image in descending aorta that generates compression of the left pulmonary artery. **C.** Angiotomography: Aortic aneurysm after coarctation.

A 9-year-old male was assessed for arterial hypertension (≥95 percentile), diagnosed 28 days before admission, and treated with angiotensin-converting enzyme inhibitors. He reported a history of fever that required hospitalization for 15 days in another hospital, with weight loss and asthenia. At clinical assessment, the following findings were noted: blood pressure in the upper right limb of 121/80 mmHg and in the ipsilateral lower limb of 94/66 mmHg for a clinical gradient of 27 mHg in systolic blood pressure, with decreased arterial pulses in the lower limbs. Auscultation revealed the presence of a GII/VI systolic murmur in the second left intercostal space. Chest X-ray showed normal cardiothoracic relationship with dense left paracardiac image. The echocardiogram showed an aortic arch with a reduced diameter at the level of the descending aorta with a gradient of 34 mmHg. Additionally, a left paracardiac tumor of 70×80 mm generated compression of the middle portion of the right pulmonary artery with a gradient of 32 mm Hg (Figure 2A and 2B). Chest computerized tomoangiography was performed and confirmed the presence of aortic coarctation with a post-stenosis saccular image that produced a compression effect toward the right pulmonary artery (Figure 2C). The patient presented significant hematemesis, for which an upper digestive endoscopy was performed and the origin of the bleeding was not identified. Varicose esophageal veins and peptic ulcer disease were excluded. Therefore, the cardiac catheterization was performed and confirmed the presence of a reduced diameter area in the descending aorta with a gradient of 34 mm Hg, after which there was saccular dilation of the descending aorta measuring 80×90 mm in diameter. Surgery was planned, but 16 hours after the catheterization, he presented a new episode of copious hematemesis with hypovolemic shock, cardiac arrest, and died.

## DISCUSSION

Currently, despite advances in the diagnosis and early treatment of aortic coarctation, it is still possible to see patients who reach adulthood without a previous diagnosis.^3,4^ Rarely, these patients may debut with symptoms of bacterial endarteritis (BE).^5^ Leninger et al. made the first report of this pathology in 1946 and, since then, there are few publications with reports of isolated cases.^6^

The occurrence of the infectious picture of BE depends on the presence of a damaged or denuded endothelium, which, in the case of aortic coarctation, is a consequence of the turbulent flow generated by the obstruction at the aortic level. This non-laminar flow damages the endothelium in the aorta area distal to the obstruction, favoring the deposit of platelets and fibrin, which constitute a substrate for the colonization of pathogenic germs during the presence of bacteremia produced mainly by Gram-positive cocci such as the *Streptococcus mitis* isolated in our first case. *Streptococcus mitis* is one of the most prevalent bacteria isolated in blood cultures after dental procedures.^7^ When bacteremia occurs and these germs circulate in the bloodstream, they adhere to the previously established substrate using multiple adhesins and forming vegetations.^3,4^ The most frequent germs involved are the same as in infective endocarditis: *Streptococcus* spp, *Staphylococcus* spp, *Salmonella* spp, *Escherichia coli*, and the Hacek group.^8^ In case 1, the bacteremia was most likely caused by gingival manipulation produced by the dental cleaning since this procedure was performed some days before the onset of fever.

In the second patient, the clinical picture was very characteristic of endarteritis, given the presence of fever in a patient with aortic coarctation, but the germ could not be isolated, possibly due to the antibiotics received prior to admission. There was no evidence of vegetation on the transthoracic echocardiogram, and we could not be certain of the diagnosis, which delayed the initiation of the respective antibiotic treatment.

Once the diagnosis of BE is established, the most feared complication is the development of false aneurysms or pseudoaneurysms, consisting of a rupture of the vascular wall that leads to an extravascular hematoma, communicating freely with the intravascular space. This characteristic differentiates it from a true aneurysm since, in the latter, dilation is surrounded by all the elements of the arterial wall. These pseudoaneurysms, called mycotic due to their bacterial origin, can be formed over a period as short as eight days, and their development and even rupture can occur despite adequate antibiotic treatment.^9,10^ In our first case, no formation of pseudoaneurysms was documented. However, in the second, although there was no histopathological confirmation, the evolution and the saccular image appreciated in the angiography and the tomography support the presence of a mycotic pseudoaneurysm in a patient with aortic coarctation without prior diagnosis and that developed a fistulous path toward the esophagus, generating a previous sentinel hemorrhage and subsequent massive bleeding that caused death due to hypovolemic shock. To date, few cases have been reported with this complication, and only nine have survived surgery, since surgical management is very laborious, requiring extensive debridement of the infected area.^11,12^

If the history of aortic coarctation was known, the initial diagnostic suspicion of BE would be clinical. However, in our cases, as in most of those described in the literature, there was no prior diagnosis of the congenital anomaly, which delays the diagnosis and the initiation of adequate treatment, increasing the risk of these vascular complications. In our two cases, the diagnosis of aortic coarctation was established 30 days after the onset of symptoms. The diagnostic opportunity was lost in the patients’ first contact with the healthcare system. Assessing pulses and measuring blood pressure in the upper and lower limbs should be considered in the initial clinical evaluation of all patients.

Schneider et al.^13^ described a clinical triad for the diagnostic suspicion of aortic coarctation with endarteritis composed by the presence of fever, hematuria, and decreased arterial pulses in the lower limbs. This triad was present in our first patient. However, for the last several years, transthoracic echocardiography has been a fundamental diagnostic tool in the initial approach to these cases, confirming the presence of the coarctation area and of vegetations or pseudoaneurysms.^4^ When transthoracic echocardiography is not conclusive, we can use the transesophageal echocardiogram looking for edema of the wall that suggests inflammation of the aorta and for the presence of vegetations or pseudoaneurysms.^4,5^ Other complementary studies, such as tomography and cardiac magnetic resonance, help to confirm the data obtained by the echocardiogram.

Management of BE is based on specific antibiotic treatment and early detection of mycotic pseudoaneurysms for surgical resolution, given the high risk of spontaneous rupture. Antibiotic treatment was started in our patients. Unfortunately, the aneurysm case evolved rapidly toward rupture. Initial clinical signs, such as hematemesis, should have alerted regarding the need of urgent surgical repair. We believe that this patient developed an aortoesophageal fistula as a drainage site for the rupture of the pseudoaneurysm, which is an even rarer variant in children and described in very few cases with endarteritis.^12^ Pseudoaneurysms surgery seeks two objectives: removing the infected part of the aorta and guaranteeing an adequate flow toward the descending and abdominal aorta.^14^ However, the dilemma remains between the indication of early surgery to reduce the chances of spontaneous rupture of the aneurysm and waiting for the absence of positive cultures to avoid reinfection. The evolution of one of our cases supports early intervention to avoid the rupture of pseudoaneurysms with fatal outcomes.

These cases illustrate that we must have a high index of clinical suspicion to be able to establish the diagnosis of aortic coarctation complicated with endarteritis and initiate appropriate antibiotic treatment, maintaining surveillance for the early detection of pseudoaneurysms and avoiding the risk of their rupture.
